# The ROMP: A Powerful Approach to Synthesize Novel pH-Sensitive Nanoparticles for Tumor Therapy

**DOI:** 10.3390/biom9020060

**Published:** 2019-02-12

**Authors:** Philippe Bertrand, Christophe Blanquart, Valérie Héroguez

**Affiliations:** 1 Institut de Chimie des Milieux et Matériaux de Poitiers, UMR CNRS 7285, 4 rue Michel Brunet, TSA 51106, B28, 86073 Poitiers CEDEX 09, France; 2 CRCINA, INSERM, Université d’Angers, Université de Nantes, 44007 Nantes, France; 3 Laboratoire de Chimie des Polymères Organiques, CNRS, UMR 5629, Bordeaux, 16 Avenue Pey-Berland, F-33607 Pessac, France

**Keywords:** epigenetic, histone deacetylases, inhibitors, nanoparticles, ring-opening metathesis polymerization, cancer, asbestos

## Abstract

Fast clearance, metabolism, and systemic toxicity are major limits for the clinical use of anti-cancer drugs. Histone deacetylase inhibitors (HDACi) present these defects, despite displaying promising anti-tumor properties on tumor cells in vitro and in in vivo models of cancer. The specific delivery of anti-cancer drugs into the tumor should improve their clinical benefit by limiting systemic toxicity and by increasing the anti-tumor effect. This paper deals with the synthesis of the polymeric nanoparticle platform, which was produced by Ring-Opening Metathesis Polymerization (ROMP), able to release anti-cancer drugs in dispersion, such as histone deacetylase inhibitors, into mesothelioma tumors. The core-shell nanoparticles (NPs) have stealth properties due to their poly(ethylene oxide) shell and can be viewed as universal nano-carriers on which any alkyne-modified anti-cancer molecule can be grafted by click chemistry. A cleavage reaction of the chemical bond between NPs and drugs through the contact of NPs with a medium presenting an acidic pH, which is typically a cancer tumor environment or an acidic intracellular compartment, induces a controlled release of the bioactive molecule in its native form. In our in vivo syngeneic model of mesothelioma, a highly selective accumulation of the particles in the tumor was obtained. The release of the drugs led to an 80% reduction of tumor weight for the best compound without toxicity. Our work demonstrates that the use of theranostic nanovectors leads to an optimized delivery of epigenetic inhibitors in tumors, which improves their anti-tumor properties in vivo.

## 1. Background 

Despite important progress being made to treat different types of cancers, acquired resistance and some forms of aggressive or less frequent cancers are still waiting for efficient strategies. The new solutions proposed to solve these problems take into account the recent advances of our knowledge in general biology, and the biology of cancer in particular, but also relative solutions for a better distribution of the compounds in the patient’s body, according to the type of cancer that needs to be treated.

In the field of biology, the last two decades have seen the dawn of the epigenetic strategy [1,2]. If genetics are currently well understood by the scientific community, a question that remains for understanding why a genome common to all our cells (genotype) is able to produce different types of cells (phenotype). This question was solved by the observation that, for the same genome, only a particular set of genes were activated in differentiated cells. This activation is the result of epigenetic mechanisms (epi: over, on top of, because of, and genêtikós: able to procreate, to father, produce).

The activation of genes is controlled, in part, by reversible post-translational modifications (PTMs) on DNA and protein histones. These PTMs involve the methylation of carbon 5 on cytosine in DNA, and oxidized versions of this methylated form (CH_2_OH, CH=O, COOH), as well as modifications on protein histones, with the most important to date being the acetylation or (poly)methylation on lysine at the N-terminal tail and the (poly)methylation of arginine residues. These various chemical modifications on DNA, or histones, are called epigenetic markers. 

This subtle mechanism of regulation can be dysregulated when the epigenetic modulators are mutated, or their level of expression has changed. This is the case with cancers, where malignant cells over-express some of these epigenetic modulators in order to reduce the expression of tumor suppressor genes. In the group of epigenetic proteins over-expressed in cancer cells, histones deacetylases (HDAC) have been thoroughly studied with the development of their inhibitors (histone deacetylase inhibitors (HDACi)), with four of them being currently approved by the Food and Drug Administration (FDA) to treat some forms of cancers. This includes the suberoyl anilide hydroxamic acid SAHA [3] (vorinostat, Zolinza^®^) and FK228 [4] (romidepsin, Istodax^®^) against cutaneous T-cell lymphoma (CTCL), belinostat [5] (Beleodaq^®^) against the refractory or regressed peripheral T-cell lymphoma (PTCL), and panobinostat (Farydak^®^) against multiple myeloma [6].

Classically, the inhibitors developed to modulate the activity of these epigenetic targets suffered from well-known limits. Clinical evaluation of HDACi has revealed that hematological and cardiac toxicity avoid the possibility of increased administrated doses, even if these compounds present a favorable toxicity profile and are well tolerated. Some other weaknesses of HDACi are their short half-life in plasma and their fast metabolism [7]. All these defects likely contribute to the poor efficiency of these molecules for treating solid tumors by limiting the fraction of active compounds reaching the tumor tissues. In order to overcome these limitations, vectorization solutions have been developed.

In the field of targeting and delivery, nanoparticle-based vectors (NPs) are often used for their intrinsic properties to accumulate in the tumor tissues, due to the enhanced permeability and retention effect (EPR) [8] described by Sato et al. in tumor blood vessels [9].

## 2. Concepts for Delivery of Epigenetic Modulators

This tumor tissue accumulation results from the deficient vascularization of tumors, which allows the transfer of NPs with a convenient size, compared to normal tissue, which does not allow this transfer. It is admitted that NPs sized in the range of 100–200 nanometers are adapted for this strategy of passive targeting. The size may vary as a function of the cell type and the NPs composition. The delivery of anti-cancer compounds with these NPs from the administration site to the tumor zone should allow for a more specific effect in the tumor. One of the problems this strategy faces is the possible entrapment by macrophages following opsonization, which can be avoided by covering the NPs with poly (ethylene oxide) (PEO) chains. The PEO chains introduce stealth properties, which reduces the recognition of exogenous NPs by these mechanisms of elimination. The selective targeting of the cancer cell can also be used to improve the delivery [10]. In this case, the NPs are also covered with biological entities, which can selectively recognize the biomarkers that are over-expressed at the cancer cell membrane surface. A third element is the mode used to release the transported drug. Enzymatic mechanisms are used, as well as the acidic tumor environment, and, within the cancer cells, the endocytosis generating acidic vesicles.

This strategy was recently applied to HDACi. It was shown that solubility, circulation time, and elimination were improved in rats after an intravenous or an oral administration of vorinostat (SAHA) when loaded in poly(ethylene oxide)-b-poly(DL-lactic acid) (PEO-b-PLA) micelles, or solid lipid nanoparticles [11,12]. Vorinostat-loaded poly(DL-lactide-co-glycolide) (PLGA) nanoparticles were demonstrated to be biocompatible, and widely distributed in the organism [13]. Additional work from the same group showed a better anti-neoplastic activity of the vorinostat-loaded PLGA nanoparticles compared to the free drugs in lung cancer cells in vitro [14]. However, the in vivo anti-tumor activity was not evaluated in these studies. This formulation was also tested in combination with chemo–radiotherapy in xenograft models of colorectal and prostate carcinomas. An improvement of the anti-tumor effect was observed when the vectored HDACi was combined with chemo–radiotherapy and compared to the free HDACi [15]. When loaded in solid lipid nanoparticles or nanostructured lipid carriers, vorinostat is more cytotoxic on breast cancer and lung cancer cell lines than the free drug [12,16]. Cholesteryl butyrate solid lipid has shown a better activity than the free butyrate on breast and promyelocytic leukemia cancer cell lines [17]. Hyaluronic acid, which is a ligand of the cancer stem cells biomarker CD44, was used for the coating of solid lipid nanoparticles loaded with vorinostat in order to target CD44 expressing cancer cells [18]. The natural class I HDACi thailandepsin A, which is a bicyclic depsipeptide, was loaded in disulphide cross-linked micelles [19]. The interest of this formulation is the disintegration of the micelles following oxidative stimuli. In a xenograft orthotopic breast cancer model, the authors showed an interesting anti-tumor effect of the vectored HDACi compared to the free FK228. However, in this work, it was not possible to appreciate the real benefit of the vectorization given that the anti-tumor effect of the free thailandepsin A was not evaluated. In another study using belinostat loaded PLGA nanoparticles modified with a cell penetrating polymer, an increased toxicity was obtained associated with a sustained hyperacetylation in bladder cancer cells compared to the free drug. This effect was confirmed in vivo with a strong decrease of the tumor volume associated with an increase of the intra-tumoral acetylation of histone H4 [20]. Similar results were obtained with vorinostat incorporated in a poly(DL-lactide-co-glycolide)/poly(ethylene glycol) copolymer using nanoprecipitation [21]. In this study, the authors obtained an interesting accumulation of the nanoparticles in the tumor tissues associated with a decrease of the tumor growth compared to bare nanoparticles. However, the benefit obtained compared to vorinostat alone was significant but modest and the subcutaneous administration route at the proximity of the tumor was not optimal for clinical transfer. The modest benefit of vorinostat vectorization in vivo on tumor growth, compared to the free molecule, was also observed in two additional studies using SAHA-loaded poly(ethylene glycol)-block-poly(caprolactone) (PEG-PCL) micelles [22] or using a self-assembly of a redox sensitive SAHA prodrug functionalized with a d-a-tocopheryl polyethylene glycol succinate [23].

Our idea for the work presented herein was to design a new delivery system using polymer-based NPs for epigenetic inhibitors with a covalent link adapted for the release at acidic pH following endocytosis [24]. The targeted application was pleural malignant mesothelioma, which is a form of lung cancer associated with asbestos exposure, and with a current first line treatment (pemetrexed + cisplatin) with low efficacy. The covalent introduction of the bioactive compounds onto the NPs was realized before the polymerization. The functionalization exploits the click chemistry between an azide and a terminal alkyne leading to an aromatic triazole ring having in our application a specific utility. Having in mind a release in acidic conditions to exploit the tumor environment and the endocytosis mechanism, a pH-responsive pro-drug system was designed to functionalize our NPs that should be internalized by endocytosis [25].

## 3. Discussion

**Scheme 1:**
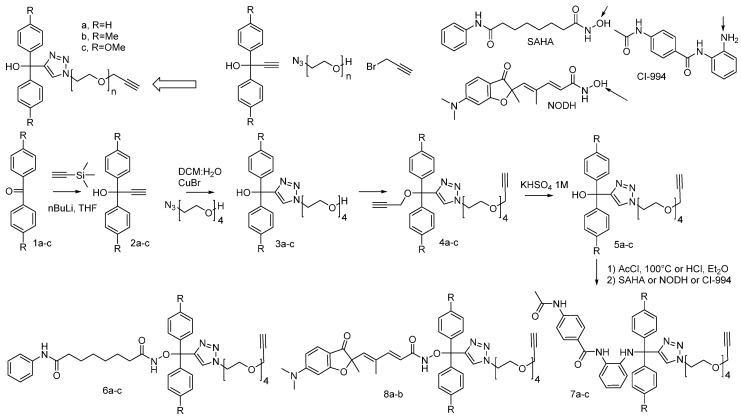
Design and summary of the synthesis of all pH-responsive pro-drugs for the three selected histone deacetylase inhibitors (HDACi).

Inspired from the trityl group (CPh_3_), which is known in the protecting group chemistry field as a pH-labile group, a new and analoguous system diaryltriazolylemethane **5** (Scheme 1) was developed to allow the modulation of the releasing kinetics at various pHs. A first objective was to obtain a half-life at pH 5 for these pro-drugs between 0.5 to 1 hour, according to the required time necessary for the maturation of acidic vesicles into lysosomes during endocytosis. The highest possible half-life at physiological pH was also necessary to avoid an early release from the vector into the blood stream.

Three HDAC inhibitors were used to validate these pro-drugs including vorinostat, tacedinaline (CI-994), and a compound prepared in Poitiers (France) known as the analogue of the trichostatin A (NODH) (Scheme 1). These molecules were covalently attached to our pH-labile clickable system through their nucleophilic function (arrow on the scheme: amine for CI-994 and hydroxamic acid for the other). This strategy may also block the known metabolic activity leading to the clearance of this type of compound.

As depicted in Scheme 1, the key aspect of this chemistry is involving a double click chemistry reaction. One click reaction was used to prepare the pH-responsive group analogous to the trityl one, and another click reaction with the remaining alkyne in compounds **6–8** was used for functionalization of the macromonomer. The pro-drugs of the three selected HDAC inhibitors SAHA, CI-994, and NODH, respectively **6a–c**, **7a–c**, and **8a–b**, were obtained in moderate yields. Fortunately, in several cases, the starting alcohols **5a–c** can be recovered from organic extracts and the inhibitors can be recovered by precipitation from the organic layers.

The behaviors of the eight pro-drugs synthesized were then validated by hydrolysis at various biologically relevant pHs: 4.3, 5.0, 6.0 for the pHs that can be found in acidic vesicles at various maturation degrees, and at pH 7.3 for the physiological and neutral pH. The results are summarized in Table 1. As one can see, the R substituent on the phenyl group is highly important for the modulation of the kinetics of the release. In the case of the two hydroxamic acid pro-drugs, the best system appeared to be the di-*p*-toluyl one (R=Me), with a half-life of 10–20 h at the moderately acidic pH of lysosomes. Other solutions were too stable or rapidly hydrolyzed. In the case of the benzamide drug, the best system was the diphenyl one (R=H). When a group with an electron donating effect was present on the phenyl rings, the benzamide pro-drugs were decomposed very quickly in a time range that limit the accurateness of the kinetic measurements. These results also indicated that the hydroxamic acid pro-drugs are more stable than the benzamide ones for the same R group. Pro-drugs of alcohols like uridine were also previously evaluated and are even more stable, where the best system is the dimethoxyphenyl **5c** for a convenient half-life of 6-7 h at pH 5 [26].

**Figure 1:**
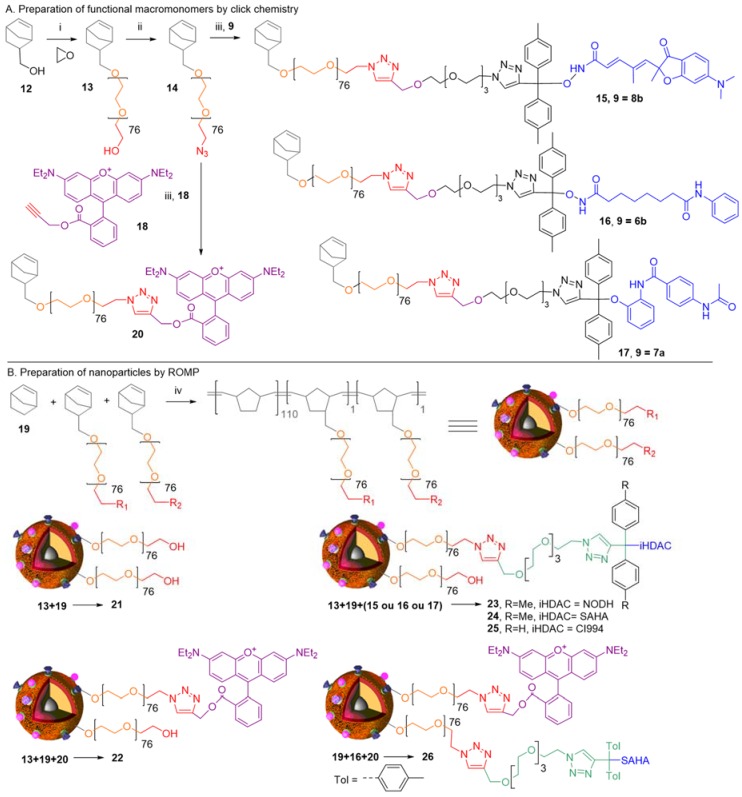
Synthesis of (**A**) the functional macro-monomers bearing the pH-responsive pro-drugs or a rhodamine fluorescent dye and (**B**) the functional polymers.

This work proposed the used of core-shell polynorbornene-based nanoparticles as a powerful strategy for multifunctional drug delivery systems (DDS). Such DDS are obtained by Ring-Opening Metathesis (ROMP) of PEO macromonomers functionalized by HDACi drugs (Figure 1). Our approach offers the advantage of flexibility in design and the poly(ethylene oxide) chains coating give stealth property required for DDS.

First, the Norborne derivative **12** (Figure 1A) is used to initiate Ring Opening Polymerization (ROP) of ethylene oxide and give access to the macromonomer NB-PEO-OH (**13**). In a second step, the hydroxyl group is converted to an azide group affording the macromonomer NB-PEO-N_3_ (**14**). In a third step, the azido macromonomer is converted in pro-drug-functionalized macromonomers by click chemistry. Macromonomers **15**, **16**, and **17** were synthesized by a coupling reaction between the macromonomer **14** and the pro-drug alkynes **8b**, **6b**, and **7a**, respectively (Scheme 1). The fluorescent macromonomer **20** was obtained following a similar route by coupling between the macromonomer **14** and the rhodamine propargylester **18**. The fluorescence of rhodamine was used to quantify the particles internalization rate in vitro and in vivo. This fluorescent dye has been chosen thanks to his long live time in in vivo, its high molar extinction coefficient (106,000 cm^−1^M^−1^ at 542.8 nm) and its high stability under acidic pH.

A wide range of NPs have been synthesized by ROM co-Polymerization of pro-drug-functionalized macromonomer (**15**, **16**, **17**) with the PEO unfunctionalized macromonomer (**13**) in dispersed media. Well-defined spherical NPs from 300 to 380 nm diameter (determined by DLS and TEM) with a hydrophilic PEO shell and a hydrophobic polynorbornene core have been obtained (Figure 1B). The NP composition was determined according to the consumed starting macro-monomers with a reported method detailed in previous research studies [27]. All NPs synthesized exhibited 99% molar of NB **19** and 1% molar of macromonomer carrying drug molecule. The nude NP **21** was prepared from NB **19** and un-functional macromonomer **13**, NP **22** by combination of **19** with **13** and fluorescent macromonomer **20**, whereas NPs **23**, **24**, and **25** are synthesized by ROMP of a mixture of NB **19** and un-functional macromonomer **13** with macromonomers **15**, **16**, and **17**, respectively. Lastly, the ROM co-Polymerization of NB **19** with macromonomers **16** and **20** together afforded NP **26**.

The synthesis of pro-drugs (Scheme 1), the determination of the hydrolysis rates of pro-drugs in mildly acidic pH [25,26], the pro-drug-functionalized macromonomer-PEO-CI994 (17), the pro-drug-functionalized macro-monomer-PEO-SAHA (16), the pro-drug-functionalized macro-monomer-PEO-NODH (15), and the synthesis of the pro-drug functionalized nanoparticles (NPs) (Figure 1), and the biological tests are developed in the supporting information part. 

## 4. Biological Effects In Vitro and In Vivo

**Figure 2:**
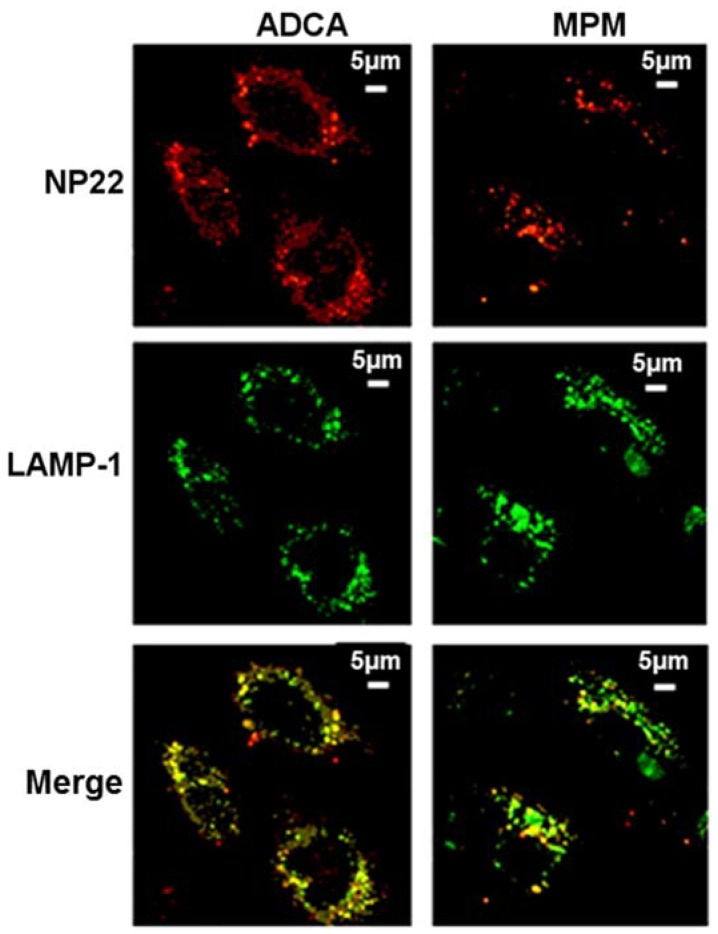
Co-localization of fluorescent NP **22** in intracellular acidic compartments of lung adenocarcinoma and mesothelioma cells. Line 1: localization of nanoparticles (NPs), line 2: LAMP-1 labelling of acidic compartments, line 3: Merge picture of line 1 and 2. Yellow color indicates a co-localization between NPs and acidic vesicles.

In the first study, we showed the internalization and the co-localization of our NPs in lysosome using rhodamine B functional NPs (**22**) and other methodologies such as flow cytometry and confocal microscopy [28]. These results demonstrated that our NPs were capable of entering into cancer cells without requiring the targeting of agents or cell penetrating peptides (CPP). The presence of NPs in the lysosomal acidic intracellular compartments (Figure 2) confirmed the validity of our approach for the functionalization of NPs with our acidic sensitive pro-drugs of HDACi.

**Figure 3:**
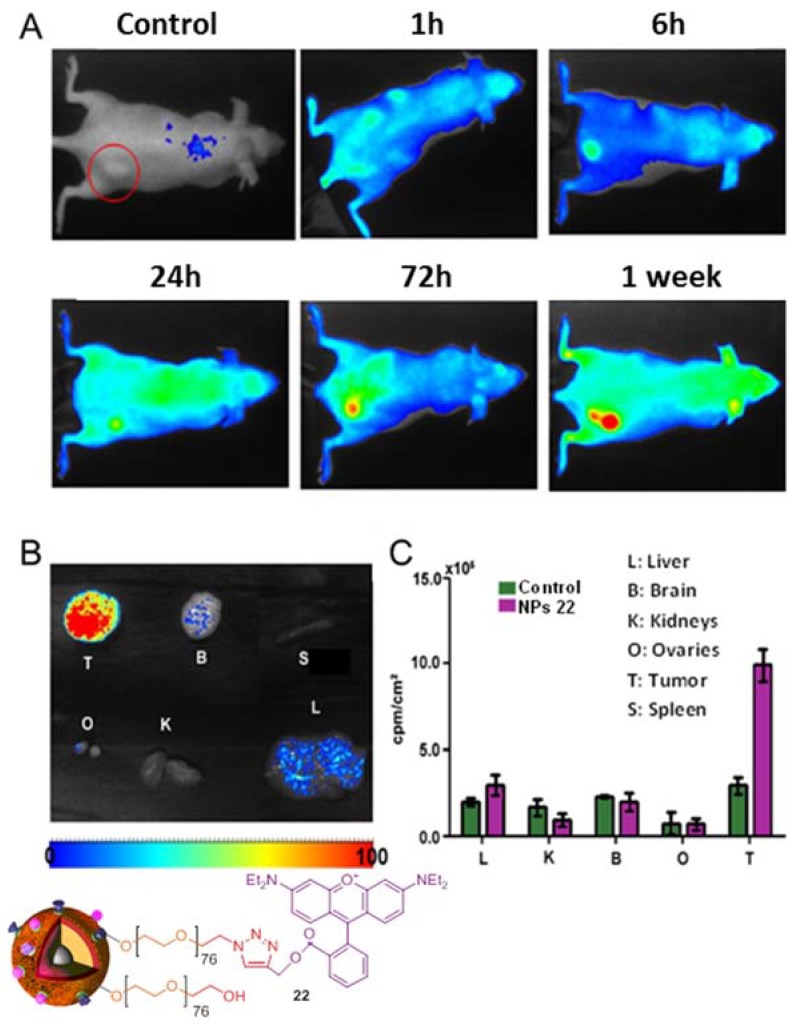
Passive targeting of xenografted tumors by NPs **22**. (**A**) Evolution of fluorescence distribution over time using whole animal imaging. (**B**) Analysis of NPs distribution on isolated organs. (**C**) Quantification of NPs accumulation in tissues over time by measuring fluorescence.

In vivo bio-distribution of our NPs was studied using two models of mesothelioma, which is a subcutaneous model and an intraperitoneal model in syngeneic mice. Rhodamine B coupled NPs and the fluorescent bio-imaging on whole animal and on isolated organs were used. The administration route was intravenous. The results obtained demonstrated a passive targeting of the tumor tissues in both models. With the subcutaneous model of mesothelioma, a clear accumulation of fluorescent NPs in tumors was observed using the whole animal imaging (Figure 3A). This particular and specific distribution was confirmed by studying the accumulation of rhodamine B fluorescence in isolated organs, to have a better sensitivity, over time [28]. A high fluorescence was obtained in tumor tissue compared with liver, spleen, kidneys, ovaries, and brain (Figure 3B,C).

**Figure 4:**
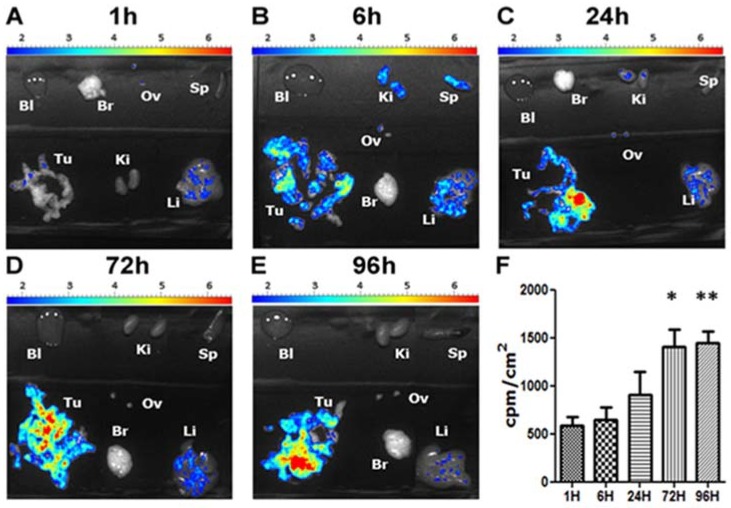
Passive targeting of diffuse peritoneal tumors by NPs **22**. (**A**–**E**) Evolution of fluorescence distribution over time using isolated organs imaging. (**F**) Quantification of NPs accumulation in tissues over time by measuring fluorescence. * *p* < 0.05 and ** *p* < 0.01. Bl: Blood, Br: Brain, Ov: Ovaries, Sp: Spleen, Tu: Tumor, Ki: Kidneys, and Li:b Liver.

Similar results were observed in the intraperitoneal model of diffuse mesothelioma after fluorescent imaging of isolated organs at different times post-injection (Figure 4) [29]. This specific passive targeting of tumor tissues by nanovectors was unusual. Thus, these two key results supported the use of our system for the delivery of HDACi in vivo and, then, to functionalize our NPs with an acidic, sensitive pro-drug of HDACi.

**Figure 5:**
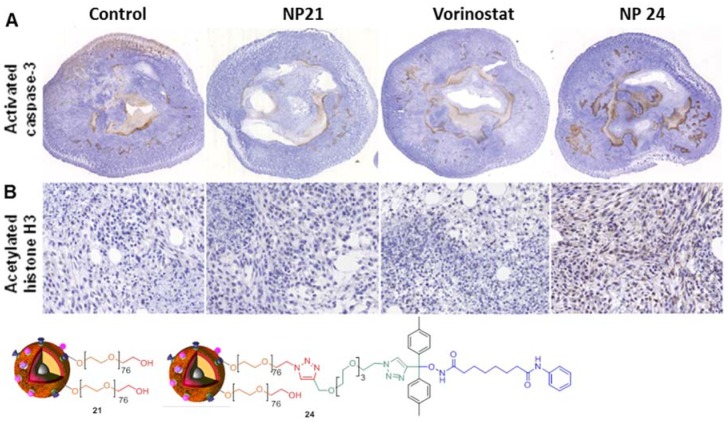
Histological analysis of tumors after treating mice with NPs **24**. Mice bearing subcutaneous AK7 tumors were injected IV with NPs **21** (160 mg/kg), with vorinostat alone (50 mg/kg), or NPs **24** (1.9 mg/kg vorinostat, 160 mg/kg polymer). Tumor tissues were analyzed using immuno-histochemistry with anti-activated capspase-3 antibody (**A**) or anti-acetylated histone H3 antibody (**B**). Blue coloration: negative labeling, brown coloration: positive labeling.

We functionalized our NPs with different pro-drugs of HDACi. In order to measure delivery of HDACi in cells, we used an assay previously described [30], which is based on the use of bioluminescence resonance energy transfer (BRET) technology. This assay allows measuring histone acetylation in living cells. In the first study, Tacedinaline (benzamide like inhibitor, Scheme 1) was used. This study using the cell viability assay showed the inhibition of HDAC and toxicity of NPs **25** on mesothelioma cell lines [31]. Similar results were obtained with NPs **24** containing vorinostat [32]. In vivo experiments showed the activity of NPs **24** in the tumor tissue, which is an increase of apoptosis (brown staining of cells) (Figure 5A) and an increase of histone H3 acetylation (brown staining of cell nuclei) (Figure 5B). In this study, we produced bifunctional NPs containing rhodamine B and pro-drug of vorinostat. The fluorescent imaging of isolated organs confirmed the specific accumulation of these bifunctional NPs in tumor tissues. However, no effect on tumor mass was observed.

All these data demonstrated that the passive targeting of tumor using NPs was very efficient. However, the absence of effect on tumor mass raised the question of the insufficient activity of the molecule used (activity at the micro-molar range) or of the insufficient functionalization level.

In order to maintain a functionalization level at 1%, NODH, which is a molecule developed at Poitiers and active at the nano-molar range, was used.

**Figure 6:**
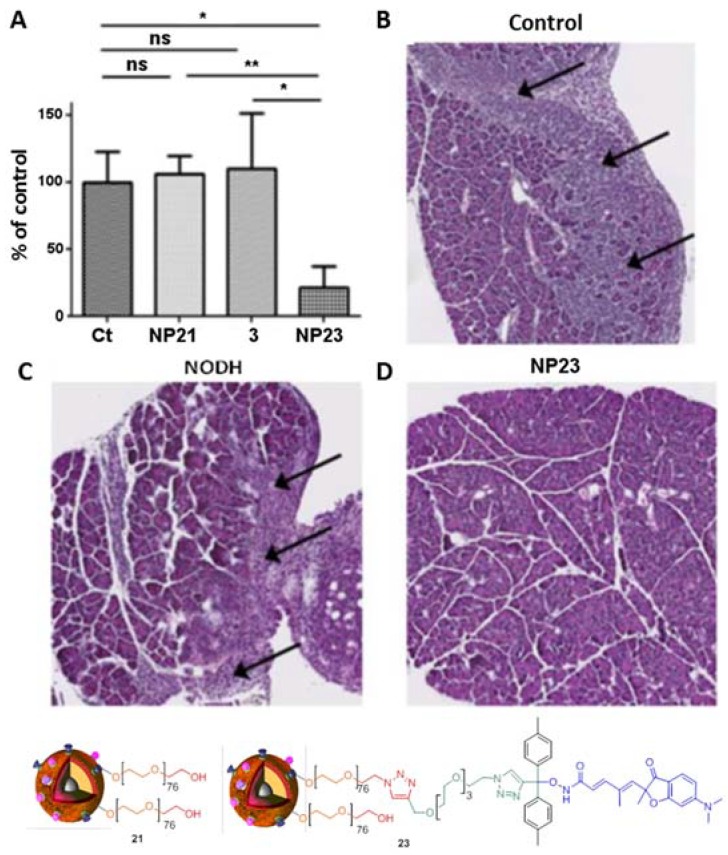
Anti-tumor activity of NPs **23**. Four groups (G1-G4) of C57Bl/6 mice bearing orthotopic AK7 tumor received intraperitoneal injection of decitabine (4 μg/g) at days 7 and 9, followed by injection IV of NPs **21** (hydroxyl NPs 80 μg/g, G2), of NODH (0.25 μg/g, G3), or NPs **23** (0.25 μg/g compound, 16 μg/g polymer, G4). G1 group received no treatment. (**A**) Graphic representation of tumor weight at the end of the protocol. (**B**–**D**) Histological pictures after HES staining of pancreas from control group G1 (**B**), group G3 treated with NODH (**C**), or group G4 treated with NPs **23** (**D**). Arrows indicate infiltration of tumor cells in pancreatic tissue. Ns, none significant. * *p* < 0.05. ** *p* < 0.01.

This compound has demonstrated improved pharmacological properties compared to vorinostat in our cell models and notably regarding resistance to cisplatin [30,33]. NPs **23** were first evaluated in vitro. We observed a decrease of cell viability associated with an increase of histone H3 acetylation, which demonstrates HDACi activity, following the treatment of cells with NPs **23**. For in vivo evaluation of NPs **23**, an intraperitoneal model of mesothelioma in immunocompetent mice was used [34]. The tumors obtained with this model are diffused and characterized by an extension to the pancreas. This model was closer to a human model. Intraperitoneal localization of mesothelioma is the second most common site of development of this disease in humans. Prior to anti-tumor effect evaluation, a bio-distribution study was performed, using NPs **22**, which confirmed the highly specific passive targeting of tumor tissues (Figure 4). Treatment of mice with NPs **23** led to a decrease of 80% of the tumor weight (Figure 6A) associated with a decrease of the pancreas invasion (Figure 6B–D), compared to the control and with free NODH conditions. While infiltration of cancer cells was observed in the control and with groups treated with free NODH (Figure 6B,C), pancreas was preserved in mice treated with NPs **23** (Figure 6D).

**Figure 7:**
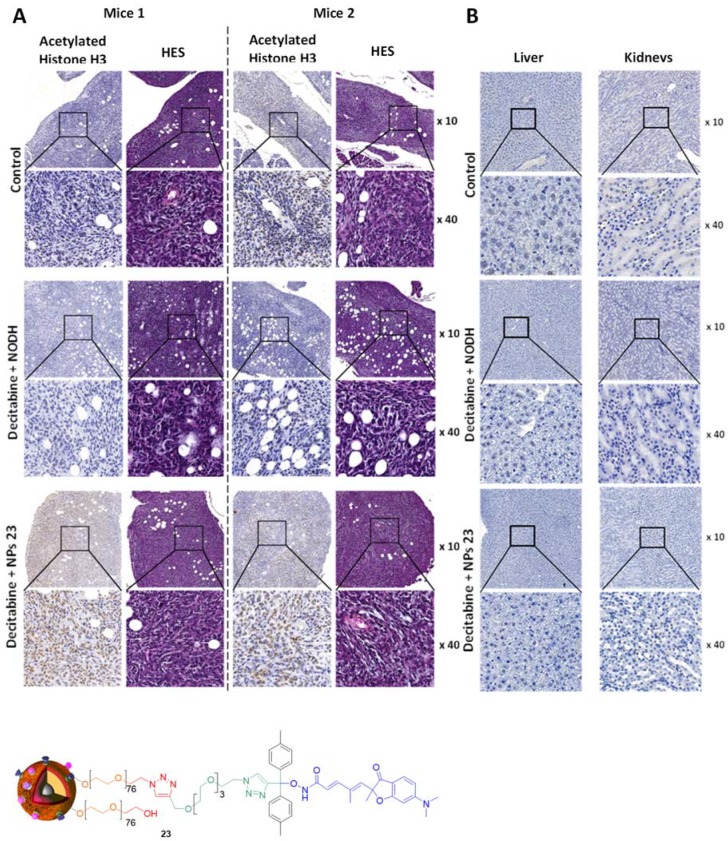
Histological analysis of tissues after treating mice with decitabine and NPs **23**. (**A**) Analysis of tumors using immuno-histochemistry with anti-acetylated histone H3 antibody and HES staining. (**B**) Analysis of liver and kidneys tissues using immuno-histochemistry with anti-acetylated histone H3 antibody. Blue coloration: negative labeling, brown coloration: positive labeling.

The anti-tumor effect obtained in the group treated with NPs **23** was associated with an increase of histone H3 acetylation in cancer cells (brown staining of cell nuclei) (Figure 7A) whereas no change in acetylation level was observed in the other analyzed organs including the liver and kidneys (Figure 7B). Additionally, no toxicity of NPs **23** on blood cells and on key organs (liver, spleen, and kidneys) was observed supporting the safety of the treatment [27].

## 5. Conclusions

All these studies demonstrate that anti-cancer treatments based only on epigenetic targets are potentially viable provided the delivery of the epigenetic inhibitors is better controlled. When compared to the other strategies developed for the delivery of epigenetic modulators, our strategy appeared highly flexible and adaptive with a lower synthetic complexity. The in vitro and in vivo experimental data show that our PEO polymer-based core-shell NPs present an excellent selectivity for the tumors without the need for specific targeting modalities, which allows a lower complexity when developing these NPs. In particular, we explored the use of vorinostat loaded norbornenyl-poly(ethylene oxide) nanoparticles in a model of mesothelioma, which is a particularly aggressive cancer related to asbestos exposure. We have used a pH sensitive pro-drug of vorinostat to avoid drug delivery during blood circulation and demonstrated a specific targeting of the tumor with their nanoparticles without haematological or systemic toxicities [25,28,32]. Whereas intra-tumoral acetylation of histone in vivo was improved with vectored vorinostat, compared to the free molecule, a poor anti-tumor effect was obtained. To improve the benefit of our system in vivo, we used an analogue of trichostatin A, NODH, which is active at nano-molar concentrations [35]. 

Various extensions are ongoing using the concepts presented in this work. Recent works have demonstrated the interest to combine or to co-vectorize HDACi with chemotherapeutic agents, the epigenetic agent serving as a sensitizer to chemotherapy. It was notably shown that particles loaded with vorinostat and bortezomib have an improved anti-tumor effect in vivo compared to the drugs when used alone [36]. This approach was applied to the co-delivery of vorinostat with paclitaxel as well as cisplatin or etoposide in different models of cancer and using different vectorization strategies [37,38,39]. 

In conclusion, the vectorization appears to be an interesting strategy to improve HDAC inhibitors activity in vivo. However, combination with classical chemotherapeutic agents needs to be considered to obtain an efficient anti-tumor activity.

## Figures and Tables

**Table 1:** Half-lives of compounds **6a–c**, **7a–c**, and **8a–b** as a function of pH.

**Compound\pH**	**3.0**	**4.3**	**5.0**	**6.0**	**7.3**
**6a**	n.d.	Stable	Stable	Stable	Stable
**6b**	n.d.	20 h	n.d.	80 h (3.3 days)	4 days
**6c**	n.d.	30 min	80 min	108 min	34 min
**7a**	n.d.	30 min	400 min	48 h	3.6 days
**7b**	n.d.	<1 min	12 min	10 min	2 h 30
**7c**	n.d.	<1 min	<5 min	<5 min	2 h 30
8a *	n.d.	stable	stable	n.d.	Stable
**8b**	3 h	13 h	21 h	n.d	3.2 days

n.d.: not determined. *: For compound **8**, based on results for compound **6**, only **8a** and **8b** were prepared.

## Supplementary Materials

Supplementary File 1
