# Supplementary material for: The ROMP: A Powerful Approach to Synthesize Novel pH-Sensitive Nanoparticles for Tumor Therapy
Source: Biomolecules. 2019 Feb 12;9(2):60. doi: 10.3390/biom9020060 (PMC6406258; doi:10.3390/biom9020060)
Supplement: Supplementary File 1 [file biomolecules-09-00060-s001.pdf]

The ROMP: A Powerful Approach to Synthesize Novel pH-Sensitive Nanoparticles for Tumor Therapy

Philippe Bertrand [1,*], Christophe Blanquart [2,*] and Valérie Héroguez [3,*]

[1] Institut de Chimie des Milieux et Matériaux de Poitiers, UMR CNRS 7285, 4 rue Michel Brunet, TSA 51106, B28, 86073 Poitiers CEDEX 09, France
[2] CRCINA, INSERM, Université d'Angers, Université de Nantes, 44007 Nantes, France
[3] Laboratoire de Chimie des Polymères Organiques, CNRS, UMR 5629, Bordeaux, 16 Avenue Pey-Berland, F-33607 Pessac, France

Supporting information

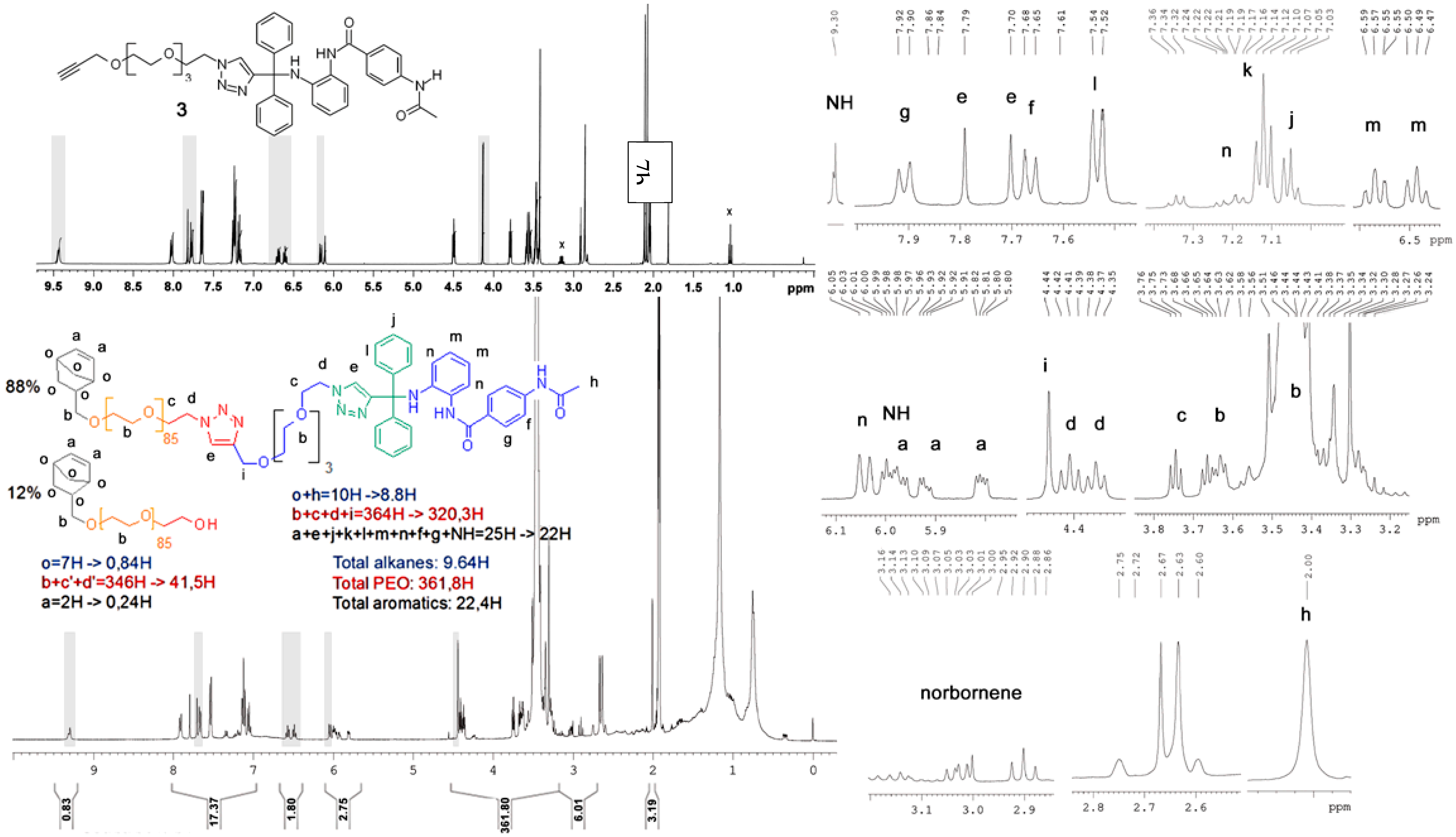

Figure S1. ^{1}HNMR in acetone D6 of clickable prodrug **7b** and macromonomer **17.**

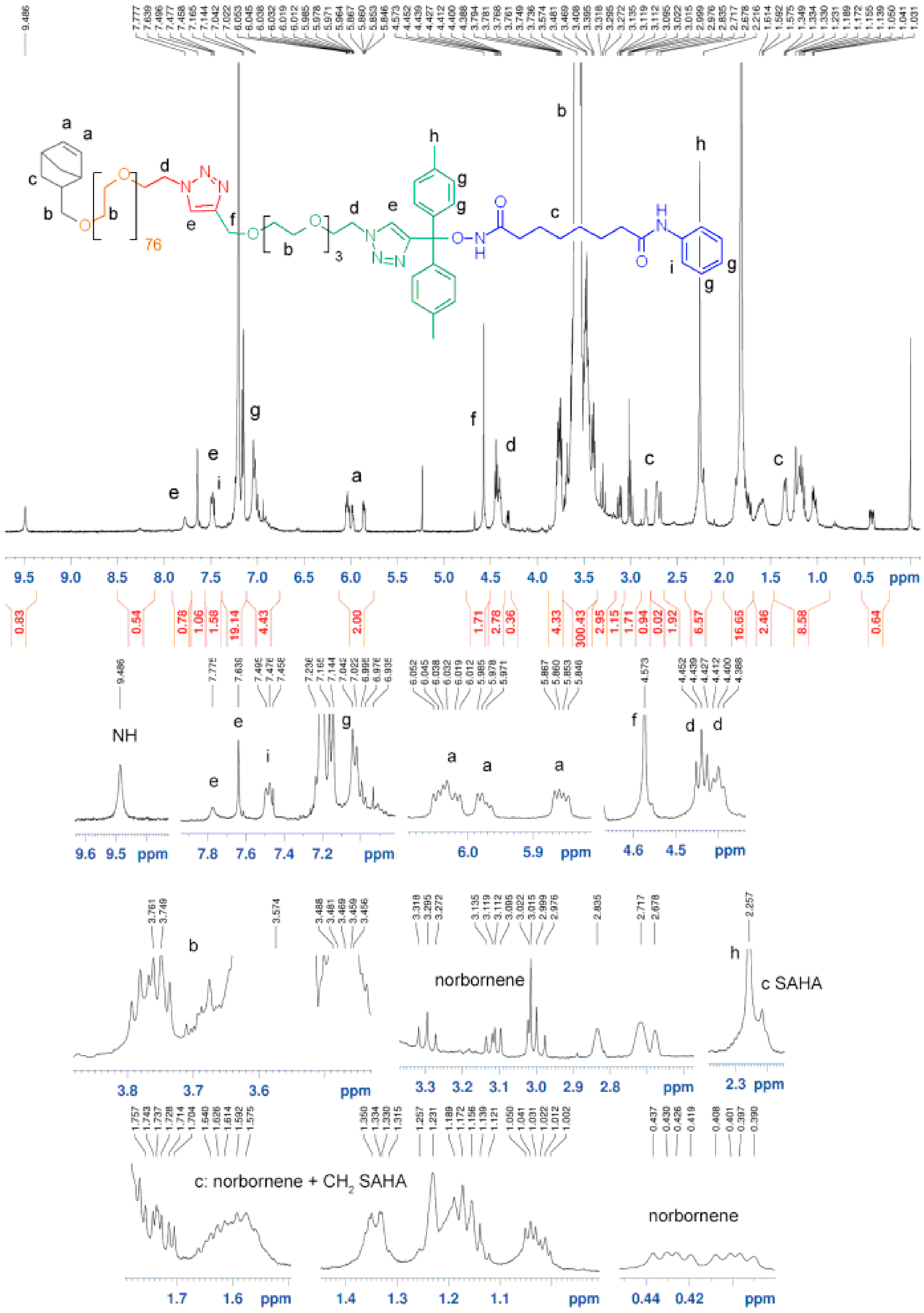

Figure S2. ^{1}HNMR in $CDCl_3$ of macromonomer **16.**

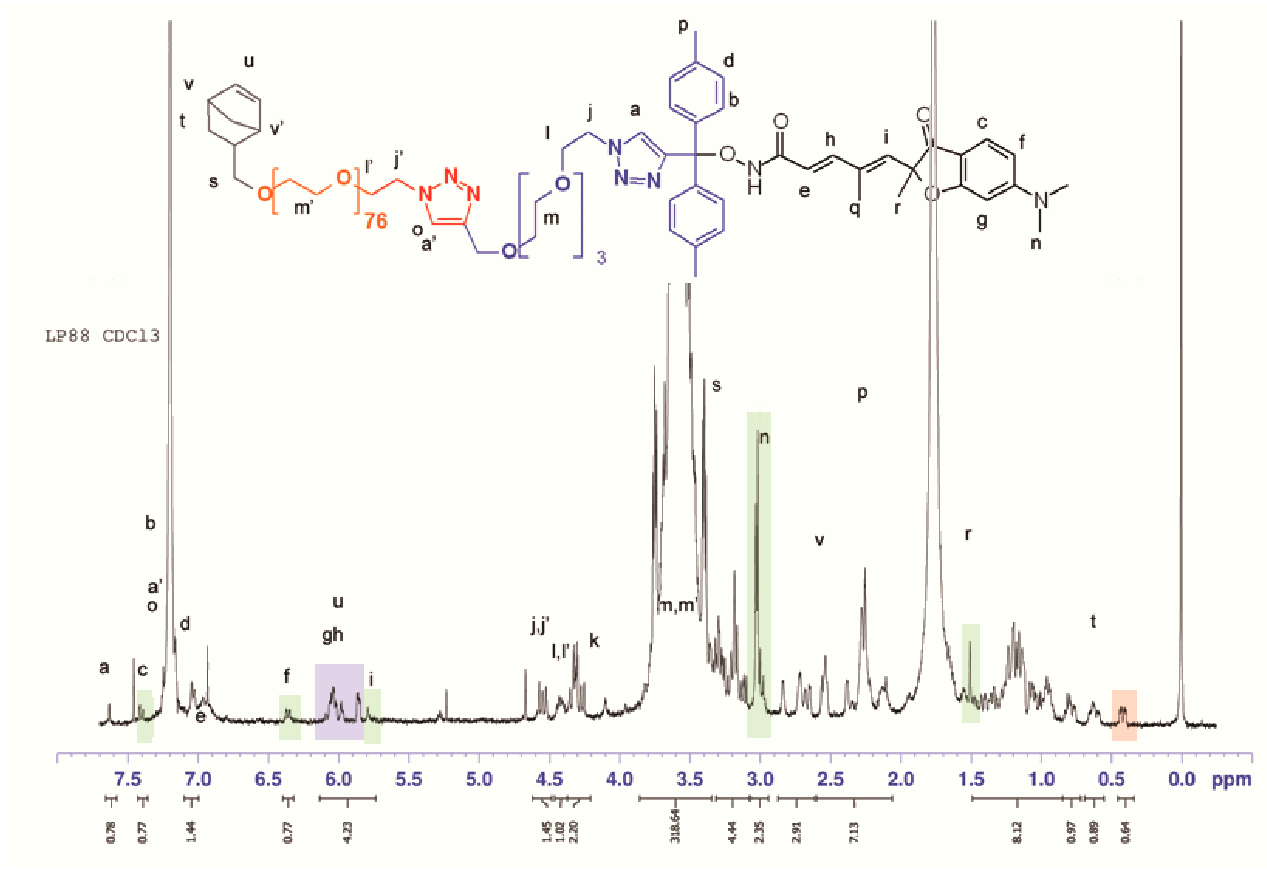

Figure S3. ^{1}HNMR in $CDCl_3$ of macromonomer **15.**

Funding data is used by funders to track result from research gra
